# Evaluating the longitudinal efficacy of platelet-rich plasma in rotator cuff surgery: a systematic review and meta-analysis

**DOI:** 10.1007/s12306-025-00906-9

**Published:** 2025-07-14

**Authors:** S. S. Gill, A. Shukla, A. Godhamgaonkar, S. R. Namireddy

**Affiliations:** 1 Imperial College London, London, UK; 2 University College London, London, UK

**Keywords:** PRP, Rotator Cuff, Rotator Cuff Repair, Shoulder, RC, RCR

## Abstract

Rotator cuff (RC) tears affect 22% of the population, increasing with age. While 48.4% are asymptomatic, 25% require surgery. Annually, over 400,000 RC repairs (RCRs) are performed in the USA, costing over $50,000 each. However, retear rates remain high (10–40%), with clinical outcomes stagnating since 1980. Platelet-rich plasma (PRP) has emerged as a potential adjunct to RCR, with its growth factors and regenerative properties offering promise for enhancing tendon healing and improving postoperative outcomes. A comprehensive search across four major databases selected studies utilising PRP in RCR. Out of 2709 studies initially identified, 22 were included, with 13 undergoing meta-analysis. The primary outcome measures were pain and functional scores, with secondary outcomes including patient-reported outcome measures (PROMs) and retear rates. PRP significantly reduced VAS scores, as a proxy of pain reduction at 6 months (MD = 0.34 [0.10, 0.59]) and at 12 months (MD = 0.24 [0.03, 0.44]) post-RCR. UCLA score improvements were significant at 3 months (MD = 2.98 [1.55, 4.40]), 6 months (MD = 1.96 [1.09, 2.83]) and 12 months (MD = 1.26 [0.39, 2.13]). Additionally, PRP significantly reduced retear rates at 24 months, with a mean risk reduction of 15.03%, indicating a substantial improvement in tendon healing. PRP offers substantial benefits in RCR, particularly in reducing pain and retear rates. However, further research is necessary due to observed heterogeneity and study biases. Future efforts should focus on incorporating PRP into clinical practice and guidelines.

**Supplementary Information:**

The online version contains supplementary material available at 10.1007/s12306-025-00906-9.

## Introduction

A rotator cuff (RC) tear is one of the most common injuries in adults, with studies suggesting that around 22% of the general population will tear their RC, with prevalence increasing with age [1–3]. Of these tears, 48.4% are likely to be asymptomatic [3]. While conservative, non-surgical, management of a rotator cuff tear is the first line of treatment, 25% of patients will require subsequent surgical intervention [4]. Every year in the USA, over 400,000 rotator cuff repairs (RCRs) are performed at an average cost of over $50,000 [5, 6]. Yet, between 10 and 40% of RCRs are likely to result in a retear [7]. Despite this, a study by McElvany et al. found that the clinical outcomes of RCR have not significantly improved since 1980 [8].

Given these challenges, the use of biologics like platelet-rich plasma (PRP), either intraoperatively or preoperatively, in patients undergoing RCRs, holds potential for improving outcomes [9, 10]. PRP is rich with fibrin, clotting factors and various growth factors alongside platelets [11]. Evidence has shown that PRP can improve tendon healing, angiogenesis and cell proliferation, thus potentially revolutionising RCR [12, 13]. PRP has been showing promise in improving the symptoms of osteoarthritis and has been shown to improve healing in numerous orthopaedic conditions when injected intra-articularly (IA) [14–17]. Currently, the surgical approach to treating RC tears primarily focuses on surgical efficacy and rehabilitation, often without the inclusion of PRP. However, PRP could be utilised as an adjunct to, or potentially as an alternative to, traditional RCR to enhance postoperative clinical and functional outcomes [18–20].

Given this potential, a comprehensive analysis with an exhaustive search is required to evaluate the use of PRP in conjunction with RCR [21]. While recent meta-analyses have explored the potential of PRP in RCR, our study aims to provide the most comprehensive review of the literature to date, offering an extensive analysis of PROMs and clinical metrics, including long-term outcomes of up to two years [19]. This systematic review aims to assess application of PRP alongside rotator cuff surgery, focusing on its impact on functional and postoperative outcomes and providing a conclusive evaluation of PRP’s role in improving RCR outcomes.

## Methods

### Protocol and registration

The intention of this study was to evaluate and review to the use of biologics in rotator cuff repairs, both intraoperatively and postoperatively. Various outcome measures were recorded at different time points and were compared to those who were not administered PRP alongside their surgical repair. This study has been registered via PROSPERO, CRD42024559543.

### Electronic search

**Fig. 1:**
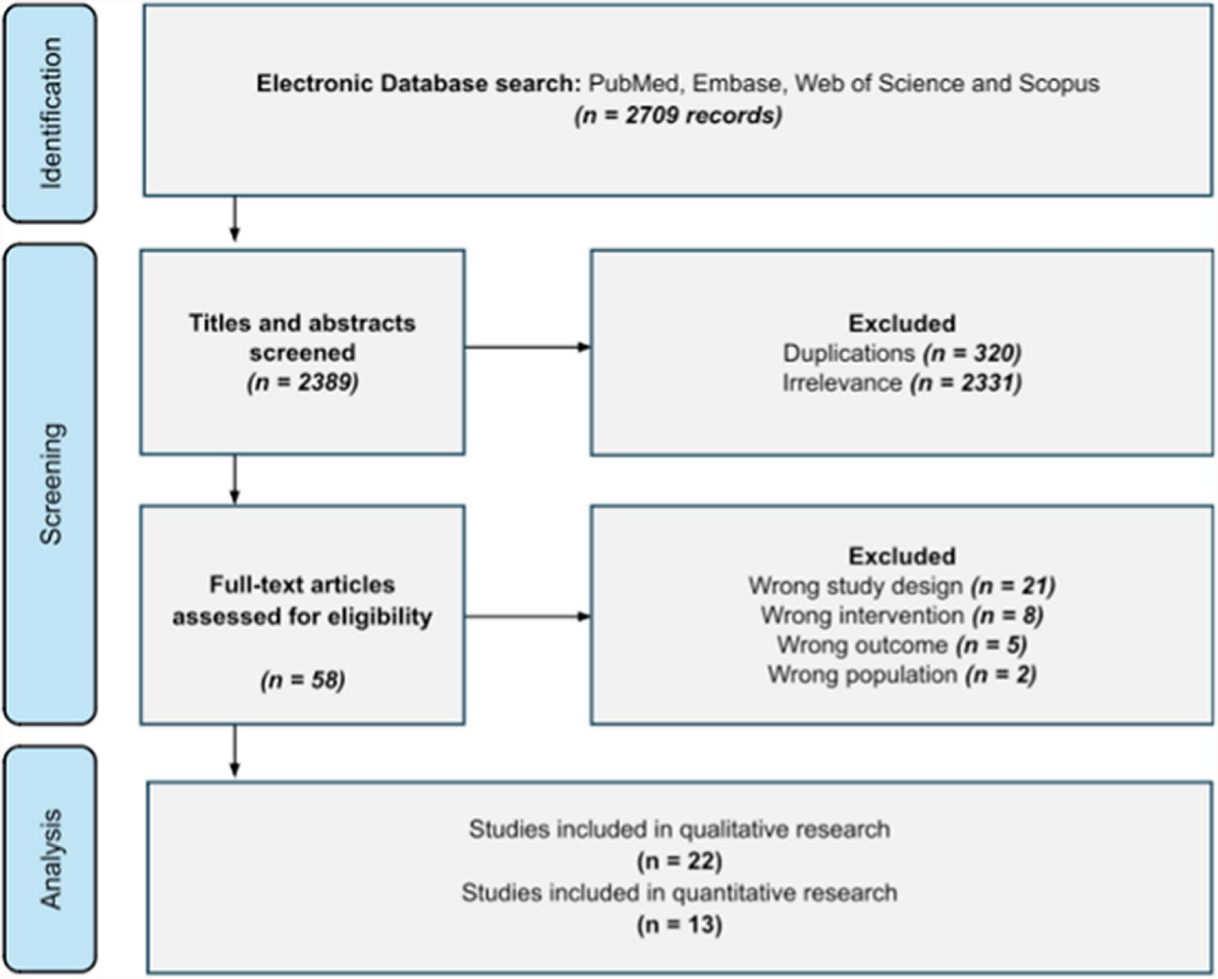
PRISMA diagram. The search yielded 1746 results from Scopus, 876 from Web of Science, 67 from MedLine and 20 from Embase

A comprehensive search strategy for randomised control trials (RCTs) was implemented in June 2024 using the numerous databases encompassing Embase, MedLine, Web of Science and Scopus. Key terms used were related to “rotator cuff surgery” and “biologics”. Only studies with full texts available were included. All papers underwent screening by two independent reviewers (SSG and AS), with a third reviewer resolving any conflicts (AG). The sources of the trials from the Cochrane Library are shown in Fig. 1 alongside a PRISMA diagram.

### Eligibility criteria and study selection

All studies that were included were RCTs with a follow-up varying from and between 1 month and 3 years, including a baseline result. Data would be collected on different outcome measures at different time points for comparison between arthroscopic rotator cuff repair alone and repair with a biologic. Both intraoperative and postoperative studies were selected; however, they would be analysed separately. All papers were assessed for their eligibility separately by two researchers (SSG and AS), and all conflicts were resolved by a third researcher (AG). Our primary outcome was pain and functional outcomes, with retear rates as our secondary outcome.

### Exclusion criteria

Papers with a follow-up time period of less than 1 month, non-randomised prospective studies, studies not conducted on humans, studies with no surgical intervention and papers without full texts available online were all excluded. Editorials, conference abstracts and study protocols were also excluded. Full criteria can be found in Table 1.

**Table 1:** Inclusion criteria

Inclusion criteria	Exclusion criteria
*Studies that focus on*	Studies more than 5 years old
PRP	
Biologics	
Rotator cuff tears	
Surgery	
Miscellaneous but relevant studies	
RCTs	Case reports
	Review articles
	Consensus statements
	Non-primary studies
	Non-randomised studies
	Cohort studies
	Editorials
	Protocols
	Theses
	Conference papers
At least one month of follow-up	Non surgical interventions
Studies applicable to adult humans	Studies written in languages other than English

### Data extraction process

When extracting the data, we manually extracted mean and standard deviation data for all data points that were available in these papers at different time points. All data were extracted into a spreadsheet. This was done by two researchers (SSG and AS). Clinical outcome data points included VAS (Visual Analogue Scale), DASH (Disabilities of the Arm, Shoulder and Hand questionnaire), UCLA (Los Angeles Shoulder Rating Scale), ASES (the American Shoulder and Elbow Surgeons Shoulder Score), WORC (Western Ontario Rotator Cuff Index) and retear rate.

### Risk of bias

Two authors independently reviewed the quality of all included studies, resolving any differences by consensus among all authors. The risk of bias in each trial was assessed using the RoB-2 tool across seven domains: randomisation method, allocation concealment, blinding of participants and personnel, blinding of outcome assessment, incomplete outcome measures, selective reporting bias and other forms of bias, categorising them as ‘low,’ ‘unclear’ or ‘high’ risk [22].

### Statistical methods

Data preparation was conducted using SPSS (IBM, USA) version 28.0.0.0. Subsequently, statistical analysis and forest plot synthesis were carried out using R software (version 4.4.3) with the meta package. Initially, a random effects model meta-analysis was performed for clinical outcome data points, including the Visual Analogue Scale (VAS), the Los Angeles Shoulder Rating Scale (UCLA), the American Shoulder and Elbow Surgeons Shoulder Score (ASES) and retear rate. All outcome variables included 95% confidence intervals and heterogeneity measured by the I^2^ test, acting as a proxy for a sensitivity analysis. An influence analysis was performed to exclude outliers. Correlation coefficients, standard errors and p-values were determined, with a *p*-value < 0.05 considered statistically significant.

## Results

### Study characteristics

Table 2 describes the studies included in the systematic review [23–44]. Table 3 outlines the data extracted from the studies included in the meta-analysis [24, 26–28, 30–33, 39, 41–43]. Table 4 describes the preparation and delivery of PRP within each study.

**Table 2:** Summary of included studies

Title	Author (Year)	Country	Results	Conclusion
Platelet-rich plasma in arthroscopic rotator cuff repair: clinical and radiological results of a prospective randomized controlled trial study at 10-year follow-up	Randelli (2022)	Italy	Follow-up satisfaction is 90% with no significant difference between PRP and control groups. Clinical outcomes are similar: CMS (PRP: 81.62 vs. Control: 77.97), UCLA (PRP: 34 vs. Control: 33) and VAS (PRP: 0.34 vs. Control: 0.70). Only ASES and SANE scores showed significant differences. Re-rupture rates are 37% for both groups (*p* = 1.00). At 2-year follow-up, new retears occurred in 6% of PRP patients and 14% of controls (*p* = 0.61)	At the 10-year follow-up, clinical and radiological outcomes are consistent between the PRP and control groups. Minor differences observed at 2 years have disappeared, and patient satisfaction remains high
Platelet-rich plasma in rotator cuff repair: a prospective randomized study	Malavolta (2014)	Brazil	Significant clinical improvement was found in the control and PRP groups (*p* < .001). At 24 months, mean UCLA scores increased (control: 13.63–32.70; PRP: 13.93 to 32.44; *p* = 0.916), Constant scores rose (control: 47.37–85.15; PRP: 46.96–84.78; *p* = 0.498) and VAS scores decreased (control: 7.00–1.15; PRP: 6.67–0.96; *p* = 0.418). A significant difference in mean UCLA scores at 12 months favoured PRP (control: 30.04 vs. PRP: 32.30, *p* = 0.046). Retear rates were similar (control: 1 complete, 4 partial; PRP: 2 partial; *p* = 0.42)	Platelet-rich plasma (PRP) did not result in better clinical outcomes or a reduced retear rate at 24-month follow-up
Platelet-rich plasma augmentation for arthroscopic rotator cuff repair: a randomized controlled trial	Castricini (2011)	Italy	No statistically significant differences in total Constant scores between the two groups following arthroscopic repair (95% CI − 3.43–3.9; *p* = 0.44) or in MRI tendon scores comparing arthroscopic repair with and without PRFM (*p* = 0.07) were reported	This study does not support using autologous PRFM to enhance double-row repair of small or medium rotator cuff tears. Results suggest PRFM may benefit larger tears, and effectiveness may vary with different PRFM preparation products
Platelet rich plasma in arthroscopic rotator cuff repair: a prospective RCT study, 2-year follow-up	Randelli (2011)	Italy	The PRP group had significantly lower pain scores at 3, 7, 14 and 30 days post-surgery (*p* < .05). At 3 months, they showed greater strength in external rotation (SER: 3 ± 1.6 kg vs. 2.1 ± 1.3 kg) and higher scores on the SST, UCLA and Constant scales (*p* < 0.05). No differences were noted at 6, 12 and 24 months, and follow-up MRIs showed no significant differences in rotator cuff healing. In grade 1 and 2 tears, SER was significantly higher in the PRP group at all follow-up points (*p* < 0.05)	Autologous PRP reduced pain in the early postoperative period, and long-term results for grade 1 and 2 tears suggest that PRP positively influences rotator cuff healing
Platelet-rich plasma supplementation in arthroscopic repair of full-thickness rotator cuff tears: a randomized clinical trial	D’Ambrosi (2016)	Italy	Preoperative VAS scores were 5.6 (PRP) and 6.4 (control) (*p* > 0.05). The PRP group had lower VAS scores at 1 week (2.5 vs. 5.3, *p* < 0.05) and 1 month (1.5 vs. 3.2, *p* < 0.05). Preoperative Constant scores were 39.95 (PRP) and 41 (control) (*p* > 0.05). After 6 months, Constant scores were 81 (PRP) and 78.5 (control) (*p* < 0.05). DASH scores were 17.4 (PRP) and 21 (control) (*p* < 0.05). No differences in ultrasound evaluations or re-ruptures were noted	PRP reduces pain in the short term, allowing for faster mobilisation and improved functionality
Platelet-rich plasma in fibrin matrix to augment rotator cuff repair: a prospective, single-blinded, randomized study with 2-year follow-up	Walsh (2018)	USA	WORC scores changed from 1257 to 139 (control) and 1106 to 99 (PRPFM) with no significant differences. The Simple Shoulder Test improved to 96% in both groups. Supraspinatus strength was 99.8% (control) and 96.3% (PRPFM). Retear rates were 19% (double-row) and 7.4% (PRPFM) at 6 months, with all being statistically insignificant	There was no benefit from PRPFM in rotator cuff repair based on WORC Index, Simple Shoulder Test and Shoulder Strength Index
Injection of leukocyte-poor platelet-rich plasma for moderate-to-large rotator cuff tears does not improve clinical outcomes but reduces retear rates and fatty infiltration: a prospective, single-blinded randomized study	Zhang (2022)	China	UCLA scores increased from 14.80 to 29.37 (control) and 13.74–30.14 (study) (*p* = 0.103). Constant scores rose from 46.56 to 86.83 (control) and 44.37 to 88.80 (study) (*p* = 0.063). VAS scores decreased from 3.22 to 0.97 (control) and 3.49–1.16 (study) (*p* = 0.41). Among 89 patients, 76 had MRIs at 24 months, with retear rates of 17.6% (study) vs. 38.1% (control) (*p* = 0.049). Goutallier grade differed postoperatively (*p* = 0.03), but not preoperatively (*p* = 0.11). No complications occurred	Lp-PRP injections reduced retear rates and improved Goutallier grade after arthroscopic rotator cuff repair, achieving minimal clinically important differences, but clinical outcomes were not significantly better than the control group
Platelet-rich plasma for arthroscopic repair of medium to large rotator cuff tears: a randomized controlled trial	Jo (2015)	Republic of Korea	No difference in Constant scores at 3 months (*p* > 0.05). The PRP group had similar VAS for pain, ROM, muscle strength, satisfaction and other scores (all *p* > 0.05), except worst pain (*p* = 0.043). The retear rate was lower in the PRP group (3.0%) veesus conventional (20.0%) (*p* = 0.032). One-year CSA change was significantly different: − 36.76 ± 45.31 mm^2^ (PRP) versus − 67.47 ± 47.26 mm^2^ (conventional) (*p* = 0.014)	PRP augmentation improved quality with lower retear rates and increased CSA of the supraspinatus but did not affect healing speed. Further studies are needed to assess PRP’s impact on healing without compromising quality
Leukocyte-poor platelet-rich plasma as an adjuvant to arthroscopic rotator cuff repair reduces the retear rate but does not improve functional outcomes: a double-blind randomized controlled trial	Rossi (2024)	Argentina	The retear rate was 15.2% (PRP) versus 34.1% (control) (*p* = 0.037). The risk ratio for ruptures with LP-PRP was 0.44 (*p* = 0.037). ASES, VAS, SANE and PSQI scores improved post-surgery (*p* < 0.001) with no significant differences between groups. Most patients exceeded the minimal clinically important difference for ASES, SANE and VAS scores	A 5-mL dose of LP-PRP significantly reduced the retear rate but did not impact postoperative pain or patient-reported outcomes of patients with rotator cuff tears that are < 3 cm via a double-row suture-bridge repair
Platelet-rich plasma for arthroscopic repair of large to massive rotator cuff tears: a randomized, single-blind, parallel-group trial	Jo (2013)	Republic of Korea	The retear rate was lower in the PRP group (20.0%) compared to the conventional group (55.6%) (*p* = 0.023). No significant differences in clinical outcomes except for overall function (*p* = 0.043). CSA change at 1-year was − 15.54 ± 94.34 mm^2^ (PRP) versus − 85.62 ± 103.57 mm^2^ (conventional) (*p* = 0.047)	PRP reduced retear rates and increased CSA in large rotator cuff repairs, suggesting potential for better long-term outcomes despite similar short-term clinical results
The effect of platelet-rich plasma on arthroscopic double-row rotator cuff repair: a clinical study with 12-month follow-up	Zhang (2016)	China	Both groups showed significant postoperative improvement (*p* < 0.05). No significant difference in overall outcome measures between groups. Retear: 30% in Group 1 versus 14% in Group 2 (*p* < 0.05)	PRP injection in arthroscopic double-row cuff repair lowered recurrence rates compared to repairs without PRP
A midterm evaluation of postoperative platelet-rich plasma injections on arthroscopic supraspinatus repair: a randomized controlled trial	Ebert (2017)	Australia	No differences in PROMs were found at midterm between groups. The PRP group had a significantly higher Constant strength score (3.3 points; *p* = 0.006). MRI scores and retear rates were similar: 66.7% of PRP patients and 64.3% of controls were Sugaya grade 1. Two control patients had symptomatic full-thickness retears within 16 weeks, while two PRP patients had partial-thickness retears later	Significant clinical improvements and high patient satisfaction followed supraspinatus repair. Maximal abduction strength was greater in the PRP group, but repeated PRP applications did not enhance tendon integrity
The effect of delayed injection of leukocyte-rich platelet-rich plasma following rotator cuff repair on patient function: a randomized double-blind controlled trial	Snow (2020)	United Kingdom	At 1 year post-operation, there were no significant differences in patient-reported outcomes or Constant scores between groups. Retear rates were similar (21% control vs. 15.3% PRP), but fatty infiltration was higher in the saline group (*p* = 0.032)	Delayed PRP application did not improve function after rotator cuff repair at 1 year
Clinical and structural evaluations of rotator cuff repair with and without added platelet-rich plasma at 5-year follow-up: a prospective randomized study	Malavolta (2018)	Brazil	At 60 months, mean UCLA scores were 32.5 (control) vs. 32.1 (PRP) (*p* = 0.992), Constant scores were 82.0 (control) versus 82.1 (PRP) (*p* = 0.699), and VAS scores were 1.4 (control) versus 1.5 (PRP) (*p* = 0.910). Both groups showed significant improvement (*p* < 0.001) with no differences at 6, 12 or 24 months. The control group had 1 full-thickness and 11 partial-thickness retears, while the PRP group had 7 partial-thickness retears, with similar overall rates (*p* = 0.203)	PRP applied during single-row repair of supraspinatus tears did not yield better clinical or structural outcomes at 60 months
Do postoperative platelet-rich plasma injections accelerate early tendon healing and functional recovery after arthroscopic supraspinatus repair? A randomized controlled trial	Wang (2015)	Australia	PRP treatment had no effect on early functional recovery, range of motion, strength or pain scores following arthroscopic supraspinatus repair. At the 16-week mark, MRI results indicated no differences in structural integrity between the PRP group (0% full-thickness retear, 23% partial tears, 77% intact) and the control group (7% full-thickness retear, 23% partial tears, 70% intact) (*p* = 0.35)	Image-guided PRP treatment does not enhance early tendon-bone healing or functional recovery post-supraspinatus repair
Platelet-rich fibrin matrix in the management of arthroscopic repair of the rotator cuff: a prospective, randomized, double-blinded study	Weber (2013)	USA	The mean surgery duration was longer in the PRFM group (83.28 ± 17.13 min) than in the control group (73.28 ± 17.18 min; *p* < 0.02). There were no significant differences in narcotic use, VAS score, SST, motion recovery or ASES, with mean ASES of 82.48 ± 8.77 (PRFM) and 82.52 ± 12.45 (control) (*p* > 0.98). Mean UCLA scores were 27.94 ± 4.98 for the PRFM group and 29.59 ± 1.68 for the control group (*p* < 0.046). Structural outcomes were affected by age and tear size, with no significant differences between the groups. No complications were reported	Platelet-rich fibrin matrix did not significantly improve perioperative morbidity, clinical outcomes or structural integrity. While longer follow-up or different PRP formulations might yield different results, early follow-up showed no significant improvements
Does application of moderately concentrated platelet-rich plasma improve clinical and structural outcome after arthroscopic repair of medium-sized to large rotator cuff tear? A randomized controlled trial	Pandey (2016)	India	The PRP group had lower visual analogue scale scores at 1, 3 and 6 months, and better Constant-Murley and UCLA scores at 12 and 24 months (*p* < 0.05). ASES scores were similar. At 24 months, retear rates were lower in the PRP group (2; 3.8%) than in controls (10; 20%; *p* = 0.01), particularly for large tears (PRP/control 1:6; *p* = 0.03). Doppler ultrasound showed increased vascularity at the PRP site at 3 months (*p* < 0.05)	Moderately concentrated PRP application improves clinical and structural outcomes in large cuff tears and enhances early vascularity around the repair site
Efficacy of intraoperative platelet-rich plasma augmentation and postoperative platelet-rich plasma booster injection for rotator cuff healing: a randomized controlled clinical trial	Liu (2021)	Republic of Korea	In patients with tears > 2 cm, the PRP group had a lower healing failure rate at 1 year compared to the control group (12.9% vs. 35.7%; *p* = 0.040). The PRP group also had lower pain scores (0.5 vs. 1.3; *p* = 0.016) and higher satisfaction scores (9.2 vs. 8.6; *p* = 0.023) than the control group. No differences were found between PRP-only and PRP booster groups in functional outcomes	Intraoperative PRP augmentation improved healing, reduced pain and increased satisfaction in patients with rotator cuff tears > 2 cm, but PRP booster injections did not offer additional benefits
Does pure platelet-rich plasma affect postoperative clinical outcomes after arthroscopic rotator cuff repair? A randomized controlled trial	Flury (2016)	Switzerland	Three months post-surgery, mean OSS was 32.9 (PRP) vs. 30.7 (control) (*p* = 0.221). No significant differences were observed in other outcomes at 6 and 24 months. Pain levels decreased similarly from day 1 to day 10 (*p* = 0.864). Recurrent supraspinatus tendon defects were 12.2% (PRP) versus 20.8% (control) (*p* = 0.295). Local adverse events occurred in 40.7% (PRP) vs. 30.5% (control) (*p* = 0.325). Smoking was a significant effect modifier	Pure PRP did not significantly improve function at 3, 6 and 24 months post-surgery compared to control. Both groups had similar pain reduction. Smoking negatively influenced PRP’s effectiveness and needs further study
Impact of platelet-rich plasma on arthroscopic repair of small- to medium-sized rotator cuff tears: a randomized controlled trial	Holtby (2016)	Canada	Both groups showed significant pain improvement within 30 days (*p* < 0.0001), with PRP reporting less pain (*p* = 0.012) and fewer painkillers (*p* = 0.026), especially from days 8 to 11. Outcome measures and ROM improved significantly after surgery (*p* < 0.0001) with no between-group differences. No differences in inflammatory markers (p > 0.05), retear rates (14% vs. 18%; *p* = 0.44) or fatty infiltration (*p* = 0.08) were observed	PRP for small- to medium-sized rotator cuff tear repair reduces short-term perioperative pain but does not significantly impact patient outcomes or repair integrity compared to control
Clinical effects of sodium hyaluronate combined with platelet-rich plasma injection on rotator cuff injury in arthroscopic repair	Zhang (2023)	China	VAS scores at 3 and 6 months post-surgery were significantly lower in the PRP group than in the control group (*p* < 0.05). Shoulder function scores (CMS, UCLA, ASES), QOL and ROM were higher in the experimental group at both follow-ups (*p* < 0.05). No significant difference in complication rates was found between the groups (*p* > 0.05)	Arthroscopic rotator cuff repair combined PRP injection effectively reduces pain, improves shoulder function and ROM and enhances QOL in patients with rotator cuff injury
A double-blinded placebo randomized controlled trial evaluating short-term efficacy of platelet-rich plasma in reducing postoperative pain after arthroscopic rotator cuff repair: a pilot study	Hak (2015)	Canada	The mean difference between the groups was not statistically significant (− 1.81; 95% CI − 4.3–1.2; *p* = 0.16). At week 6, EQ-5D, WORC and DASH scores showed no significant differences (*p* = 0.5, 0.99, 0.9). No revision surgeries were needed, with 4 adverse events reported, 3 of which were in the PRP group	No statistical differences in outcomes, when PRP is used in arthroscopically repaired rotator cuff, were reported

**Table 3:** Extracted data

Author (Year)	N (control/PRP+)	Mean age (control/PRP+)	VAS	DASH	UCLA	ASES	Retear rate
Malavolta et al. (2014)	27/27	54.1/55.3	–		–		–
Randelli et al. (2011)	27/26	59.5/61.6	–		–		–
D’Ambrosi et al. (2016)	20/20	62.0/57.9	–	–			
Walsh et al. (2018)	44/28	54.9/56.9					–
Jo et al. (2015	37/37	60.9/60.1	–		–	–	–
Rossi et al. (2024)	48/48	56.1/56.2	–			–	–
Jo et al. (2013)	24/24	61.9/64.2	–	–	–	–	–
Zhang et al. (2016)	30/30	57.2/56.9	–	–			–
Pandey et al. (2016)	50/52	54.1/54.8	–		–	–	–
Liu et al. (2021)	48/48	63.3/63.0	–			–	
Flury et al. (2016)	60/60	58.9/57.8		–		–	–
Holtby et al. (2016)	41/41	59.0/59.0	–			–	–
Zhang et al. (2023)	46/46	54.2/56.2	–		–	–	–

Table 3 describes the available data that was extracted from the studies included in the meta-analysis. N-dash (–) columns indicate data being extracted in these categories

**Table 4:** Preparation and application of PRP

Title	Author (Year)	Preparation	Application
Platelet-rich plasma in arthroscopic rotator cuff repair: clinical and radiological results of a prospective randomized controlled trial study at 10-year follow-up	Randelli (2022)	6 ml autologous thrombin, which was mixed with PRP using a 1:5 ratio of 10% calcium chloride solution	Subacromial
Platelet-rich plasma in rotator cuff repair: a prospective randomized study	Malavolta (2014)	24.6 mL was applied, comprising 20 mL of PRP, 3 mL of thrombin and 1.6 mL of calcium chloride	Subacromial
Platelet-rich plasma augmentation for arthroscopic rotator cuff repair: a randomized controlled trial	Castricini (2011)	Unclear	Unclear
Platelet rich plasma in arthroscopic rotator cuff repair: a prospective RCT study, 2-year follow-up	Randelli (2011)	6 ml PRP, with 1:5 of 10% calcium chloride	Unclear
Platelet-rich plasma supplementation in arthroscopic repair of full-thickness rotator cuff tears: a randomized clinical trial	D’Ambrosi (2016)	6 ml PRP	Unclear
Platelet-rich plasma in fibrin matrix to augment rotator cuff repair: a prospective, single-blinded, randomized study with 2-year follow-up	Walsh (2018)	Unclear	Unclear
Injection of leukocyte-poor platelet-rich plasma for moderate-to-large rotator cuff tears does not improve clinical outcomes but reduces retear rates and fatty infiltration: a prospective, single-blinded randomized study	Zhang (2022)	3 mL of Lp-PRP and 0.8 mL of calcium chloride (10%)	Unclear
Platelet-rich plasma for arthroscopic repair of medium to large rotator cuff tears: a randomized controlled trial	Jo (2015)	0.3 mL of 10% calcium gluconate was added to 3 mL of PRP	Unclear
Leukocyte-poor platelet-rich plasma as an adjuvant to arthroscopic rotator cuff repair reduces the retear rate but does not improve functional outcomes: a double-blind randomized controlled trial	Rossi (2024)	5 mL of PRP	Subacromial
Platelet-rich plasma for arthroscopic repair of large to massive rotator cuff tears: a randomized, single-blind, parallel-group trial	Jo (2013)	0.3 mL of 10% calcium gluconate was added to 3 mL of PRP	Suture
The effect of platelet-rich plasma on arthroscopic double-row rotator cuff repair: a clinical study with 12-month follow-up	Zhang (2016)	Unclear	Subacromial
A midterm evaluation of postoperative platelet-rich plasma injections on arthroscopic supraspinatus repair: a randomized controlled trial	Ebert (2017)	Approximately 2–4 mL PRP	Suture
The effect of delayed injection of leukocyte-rich platelet-rich plasma following rotator cuff repair on patient function: a randomized double-blind controlled trial	Snow (2020)	6 mL of LR-PRP	Subacromial
Clinical and structural evaluations of rotator cuff repair with and without added platelet-rich plasma at 5-year follow-up: a prospective randomized study	Malavolta (2018)	10 mL of PRP and 0.4 mL of 10% calcium chloride	Subacromial
Do postoperative platelet-rich plasma injections accelerate early tendon healing and functional recovery after arthroscopic supraspinatus repair? A randomized controlled trial	Wang (2015)	Approximately 2–4 mL PRP	Subacromial
Platelet-rich fibrin matrix in the management of arthroscopic repair of the rotator cuff: a prospective, randomized, double-blinded study	Weber (2013)	Unclear	Unclear
Does application of moderately concentrated platelet-rich plasma improve clinical and structural outcome after arthroscopic repair of medium-sized to large rotator cuff tear? A randomized controlled trial	Pandey (2016)	8 mL PRP mixed with 1.6 mL 10% calcium chloride (5:1 ratio)	Subacromial
Efficacy of intraoperative platelet-rich plasma augmentation and postoperative platelet-rich plasma booster injection for rotator cuff healing: a randomized controlled clinical trial	Liu (2021)	3 mL PRP mixed with 0.3 mL 10% calcium gluconate	Subacromial
Does pure platelet-rich plasma affect postoperative clinical outcomes after arthroscopic rotator cuff repair? A randomized controlled trial	Flury (2016)	4 ml PRP	Subacromial
Impact of platelet-rich plasma on arthroscopic repair of small- to medium-sized rotator cuff tears: a randomized controlled trial	Holtby (2016)	7 ml PRP	Subacromial
Clinical effects of sodium hyaluronate combined with platelet-rich plasma injection on rotator cuff injury in arthroscopic repair	Zhang (2023)	Unclear	Subacromial
A double-blinded placebo randomized controlled trial evaluating short-term efficacy of platelet-rich plasma in reducing postoperative pain after arthroscopic rotator cuff repair: a pilot study	Hak (2015)	9–13,5 ml PRP	Directly into RC tendon then subacromial

RC, rotator cuff; PRP, platelet-rich plasma

### Risk of bias assessment

**Fig. 2:**
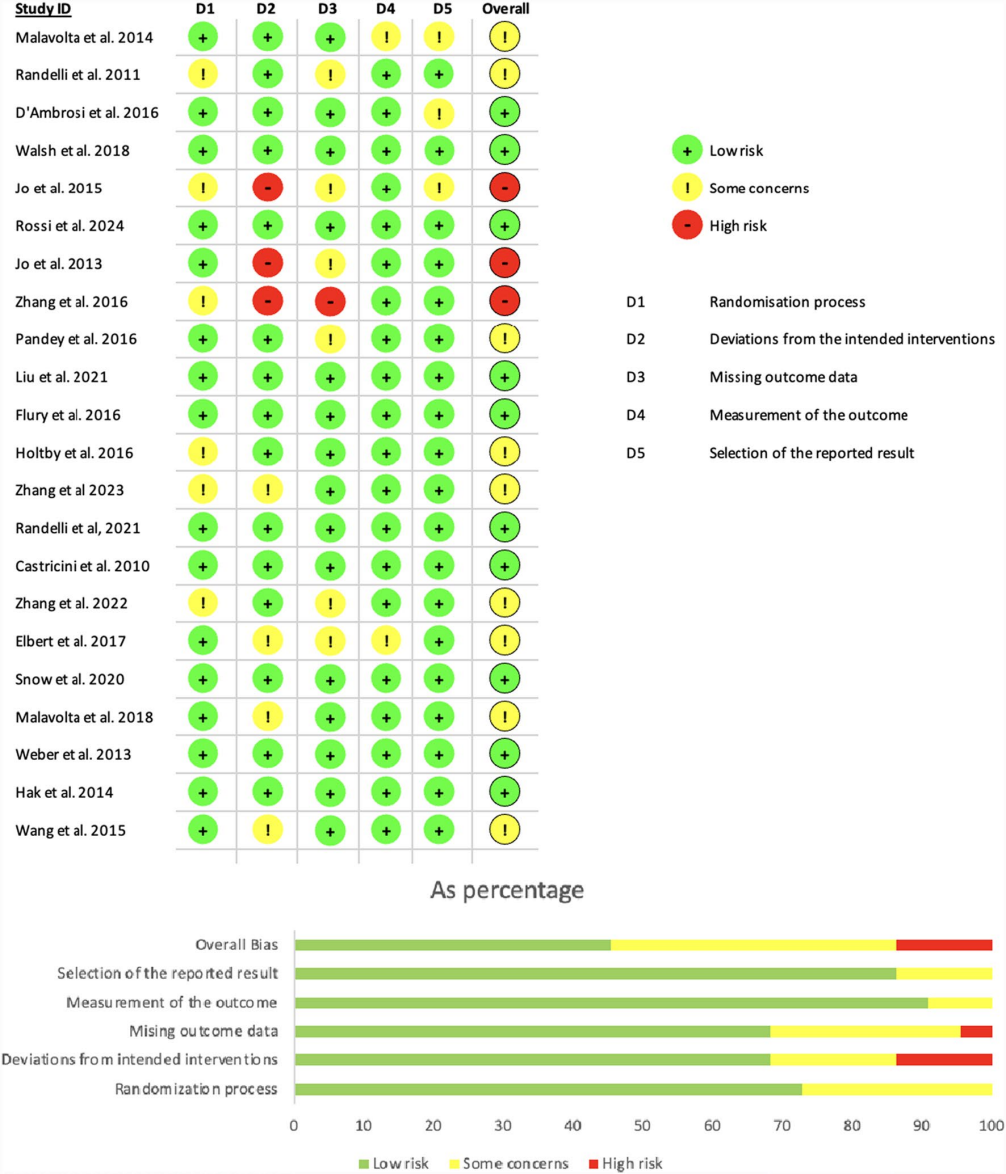
Risk of bias assessment of all papers used in qualitative and quantitative analysis using the RoB2 tool

The Risk of Bias (RoB) assessment revealed that the majority of the studies exhibited a low risk of bias (see Fig. 2) [25–28, 31, 35, 38, 40, 41, 44]. In terms of randomisation, six studies (27.3%) had 'some concerns', though none were rated as high risk [26, 29, 30, 33, 42, 43]. However, three studies (13.6%) demonstrated high risk for deviation from intended interventions [30, 32, 33], while one study (4.55%) had missing outcome data [33]. These two categories presented the highest risk of bias. Conversely, minimal bias was detected in the measurement of outcomes and selection of reported results, with no studies classified as high risk in these areas. Overall, three studies (13.6%) had high overall risk of bias [30, 32, 33], nine studies (40.9%) had some concerns associated with overall risk of bias [23, 24, 29, 34, 36, 37, 39, 42, 43], and ten studies (45.5%) had a high risk of bias [25–28, 31, 35, 38, 40, 41, 44].

### Visual analogue scale—pain

**Fig. 3:**
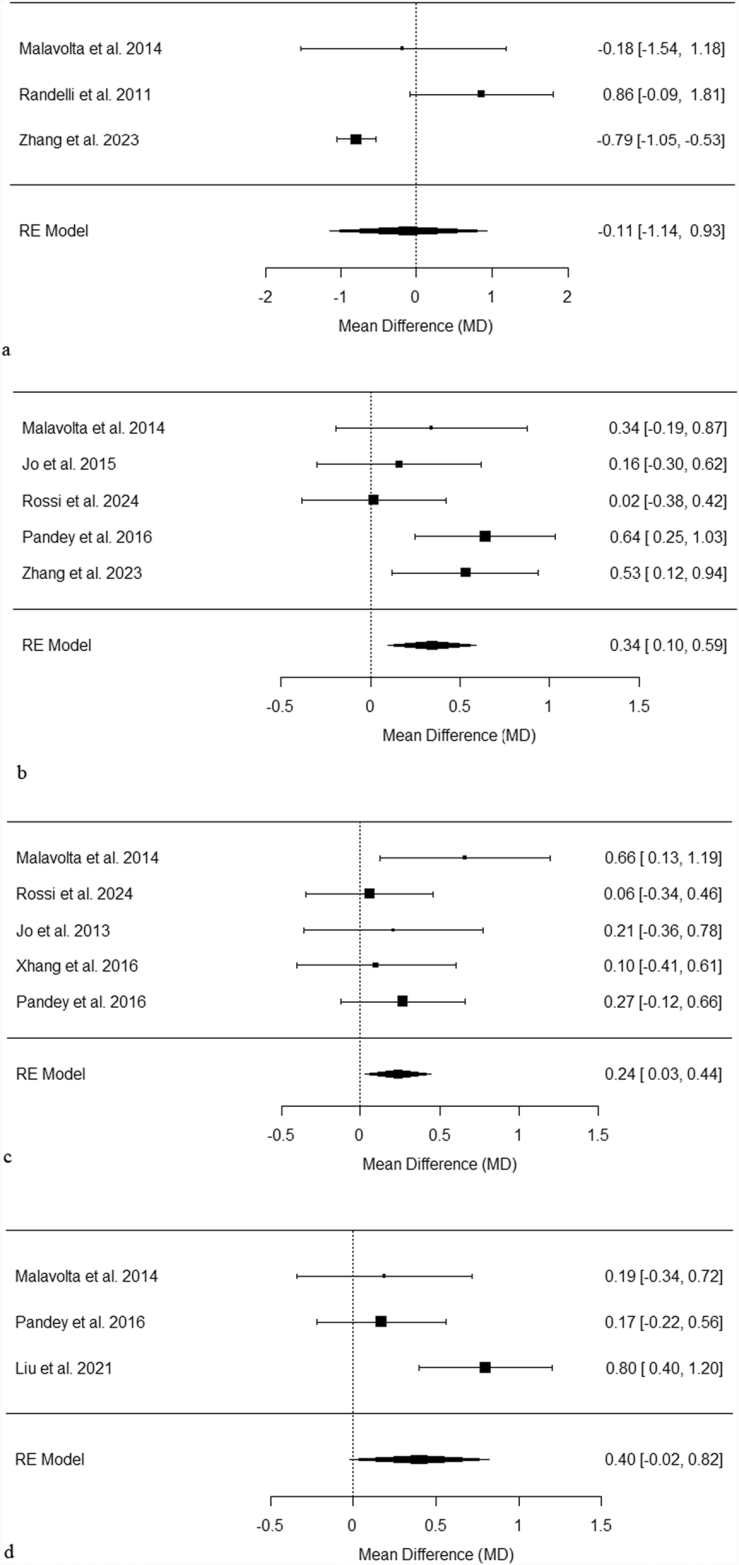
**a** Forest plot of pooled VAS scores at 3 months post-surgery. This plot displays the mean differences in VAS scores across different studies, each represented by a black square proportional to the weight of the study. Horizontal lines show the 95% confidence intervals for each mean difference. The pooled effect from the random effects model is depicted by a black diamond, indicating the combined mean difference and its 95% confidence interval. **b** Forest plot of pooled VAS scores at 6 months post-surgery. This plot displays the mean differences in VAS scores across different studies, each represented by a black square proportional to the weight of the study. Horizontal lines show the 95% confidence intervals for each mean difference. The pooled effect from the random effects model is depicted by a black diamond, indicating the combined mean difference and its 95% confidence interval. **c** Forest plot of pooled VAS scores at 12 months post-surgery. This plot displays the mean differences in VAS scores across several studies, each represented by a black square proportional to the weight of the study. Horizontal lines show the 95% confidence intervals for each mean difference. The pooled effect from the random effects model is depicted by a black diamond, indicating the combined mean difference and its 95% confidence interval. **d** Forest plot of pooled VAS scores at 24 months post-surgery. This plot displays the mean differences in VAS scores across several studies, each represented by a black square proportional to the weight of the study. Horizontal lines show the 95% confidence intervals for each mean difference. The pooled effect from the random effects model is depicted by a black diamond, indicating the combined mean difference and its 95% confidence interval

Figure 3a presents the mean differences in Visual Analogue Scale (VAS) scores at 3 months post-surgery across several studies [23, 24, 44]. The observed mean differences range from 0.79 to 0.86, with none of the studies demonstrating a significant improvement in VAS scores with the addition of PRP injection to surgery compared to surgery alone. Notably, Zhang et al. significantly favoured the non-PRP group [44]. The pooled mean difference is − 0.11 [− 1.14, 0.93], with moderate heterogeneity (I^2^ = 78.81%, *p* < 0.01).

Figure 3b shows the mean differences in VAS scores at 6 months post-surgery across various studies [24, 30, 31, 39, 43]. The differences range from 0.02 to 0.64, with two out of six studies indicating a significant improvement in VAS scores from combining PRP injection with surgery compared to surgery alone [39, 43]. The pooled mean difference is 0.34 [0.10, 0.57], with moderate heterogeneity (I^2^ = 37.18%, *p* = 0.19).

Figure 3c depicts the mean differences in VAS scores at 12 months post-surgery across several studies [24, 31–33, 39]. The observed differences range from 0.06 to 0.66. One of the five studies showed a significant improvement in VAS scores with PRP injection in addition to surgery compared to surgery alone [24]. The pooled mean difference is 0.24 [0.03, 0.44], with low heterogeneity (I^2^ = 7.26%, *p* = 0.47).

Figure 3d illustrates the mean differences in VAS scores at 24 months post-surgery across multiple studies [24, 39, 40]. The mean differences observed range from 0.79 to 0.86, with none of the studies showing a significant improvement in VAS scores when PRP injection was added to surgery compared to surgery alone. Liu et al. significantly favoured the non-PRP group [40]. The pooled mean difference is 0.40 [− 0.02, 0.82], with intermediate heterogeneity (I^2^ = 64.07%, *p* = 0.06).

### University of California–Los Angeles shoulder rating—functionality

**Fig. 4:**
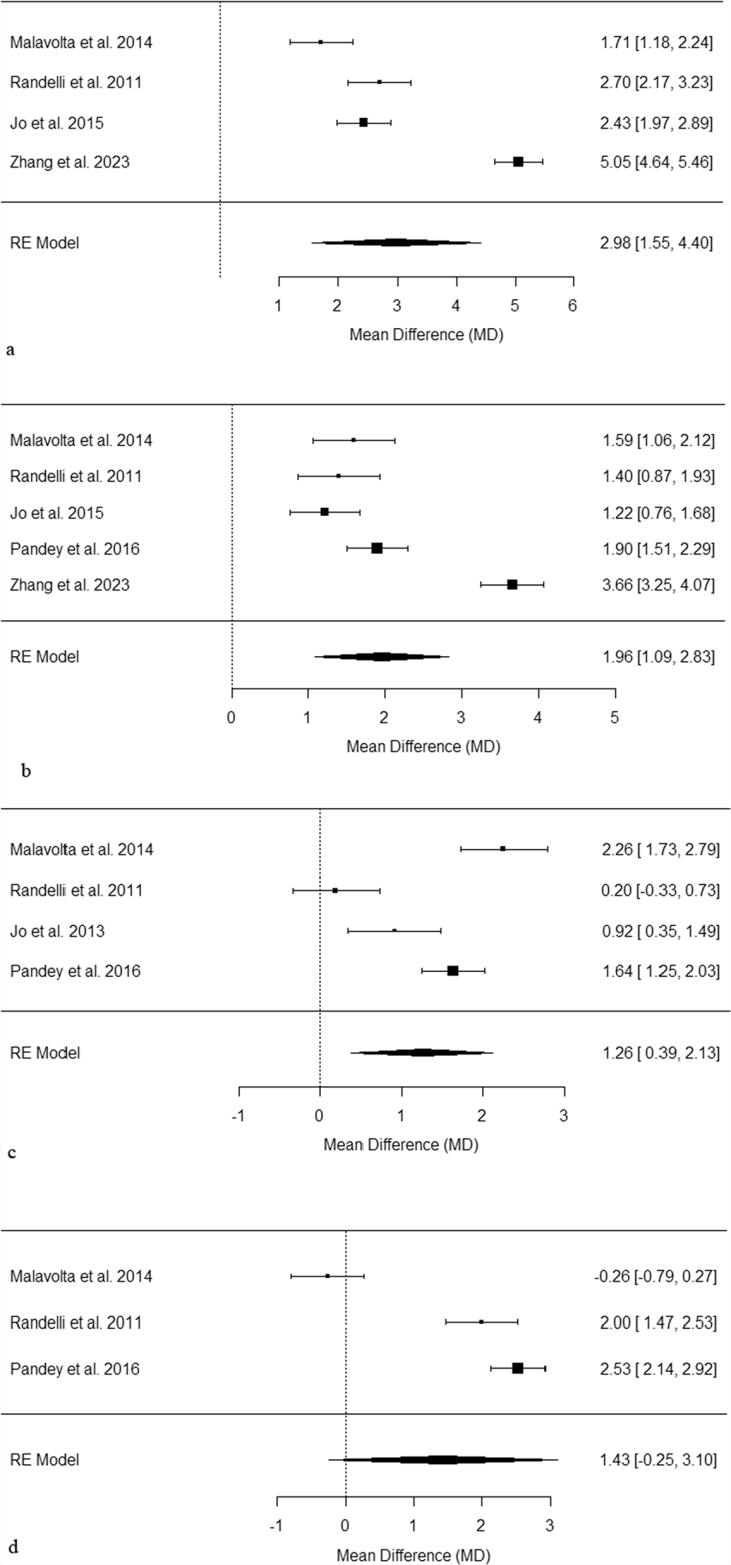
**a** Forest plot of pooled UCLA scores at 3 months post-surgery. This plot displays the mean differences in UCLA scores across several studies, each represented by a black square proportional to the weight of the study. Horizontal lines show the 95% confidence intervals for each mean difference. The pooled effect from the random effects model is depicted by a black diamond, indicating the combined mean difference and its 95% confidence interval. **b** Forest plot of pooled UCLA scores at 6 months post-surgery. This plot displays the mean differences in UCLA scores across several studies, each represented by a black square proportional to the weight of the study. Horizontal lines show the 95% confidence intervals for each mean difference. The pooled effect from the random effects model is depicted by a black diamond, indicating the combined mean difference and its 95% confidence interval. **c** Forest plot of pooled UCLA scores at 12 months post-surgery. This plot displays the mean differences in UCLA scores across several studies, each represented by a black square proportional to the weight of the study. Horizontal lines show the 95% confidence intervals for each mean difference. The pooled effect from the random effects model is depicted by a black diamond, indicating the combined mean difference and its 95% confidence interval. **d** Forest plot of pooled UCLA scores at 24 months post-surgery. This plot displays the mean differences in UCLA scores across several studies, each represented by a black square proportional to the weight of the study. Horizontal lines show the 95% confidence intervals for each mean difference. The pooled effect from the random effects model is depicted by a black diamond, indicating the combined mean difference and its 95% confidence interval

Figure 4a analyses the UCLA scores at 3 months post-surgery. The mean differences observed range from 1.71 to 5.05, reflecting varied improvements in shoulder function. All four studies demonstrated significant improvements, with the most pronounced effects seen in Zhang et al. 2023 [24, 26, 30, 43]. The pooled mean difference is 2.98 [1.55, 4.40], with high heterogeneity (I^2^ = 97.18%, *p* < 0.001).

Figure 4b presents UCLA scores at 6 months post-surgery. The observed mean differences span from 1.22 to 3.66, indicating a range of patient outcomes in shoulder function recovery. All five studies reported significant improvements with additional PRP [24, 26, 30, 39, 43]. The pooled mean difference is 1.96 [1.09, 2.83], with high heterogeneity (I^2^ = 94.5%, *p* < 0.001).

Figure 4c reviews UCLA scores at 12 months post-surgery. Mean differences vary from 0.20 to 2.26 [24, 26, 32, 39]. Three out of four studies showed significant results when comparing outcomes between surgery and surgery with PRP [24, 32, 39]. The pooled mean difference is 1.26 [0.39, 2.13], with high heterogeneity (I^2^ = 91.72%, *p* < 0.001).

Figure 4d analyses UCLA scores at 24 months post-surgery. The range of mean differences is from − 0.26 to 2.53 [24, 26, 39]. Two of the three studies showed significant improvement when using PRP alongside surgery [26, 39]. The pooled mean difference is 1.43 [− 0.25, 3.10], with high heterogeneity (I^2^ = 97.23%, *p* < 0.001).

### American shoulder and elbow surgeons − score pain + function

**Fig. 5:**
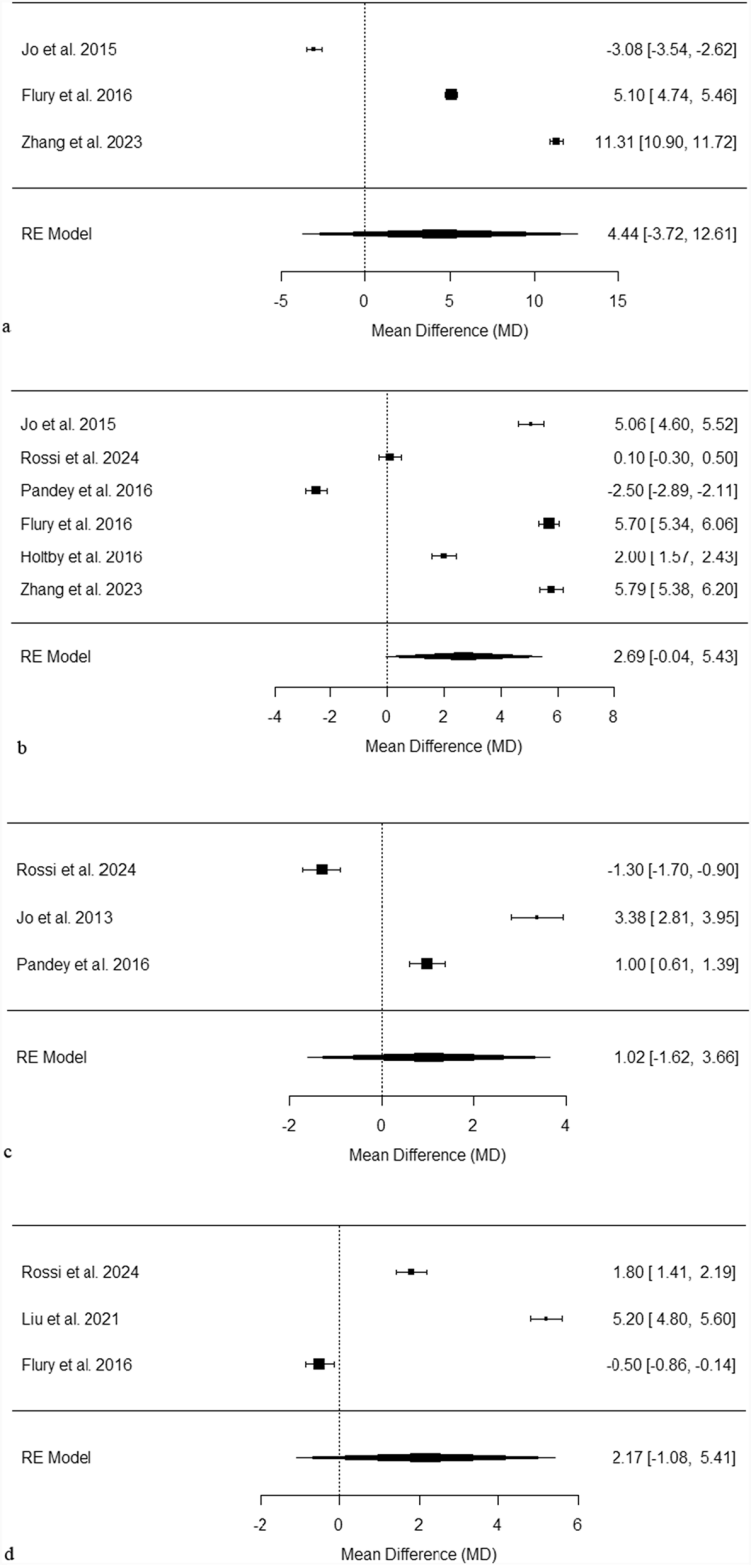
**a** Forest plot of pooled ASES scores at 3 months post-surgery. This plot displays the mean differences in ASES scores across several studies, each represented by a black square proportional to the weight of the study. Horizontal lines show the 95% confidence intervals for each mean difference. The pooled effect from the random effects model is depicted by a black diamond, indicating the combined mean difference and its 95% confidence interval. **b** Forest plot of pooled ASES scores at 6 months post-surgery. This plot displays the mean differences in ASES scores across several studies, each represented by a black square proportional to the weight of the study. Horizontal lines show the 95% confidence intervals for each mean difference. The pooled effect from the random effects model is depicted by a black diamond, indicating the combined mean difference and its 95% confidence interval. **c** Forest plot of pooled ASES scores at 12 months post-surgery. This plot displays the mean differences in ASES scores across several studies, each represented by a black square proportional to the weight of the study. Horizontal lines show the 95% confidence intervals for each mean difference. The pooled effect from the random effects model is depicted by a black diamond, indicating the combined mean difference and its 95% confidence interval. **d** Forest plot of pooled ASES scores at 24 months post-surgery. This plot displays the mean differences in ASES scores across several studies, each represented by a black square proportional to the weight of the study. Horizontal lines show the 95% confidence intervals for each mean difference. The pooled effect from the random effects model is depicted by a black diamond, indicating the combined mean difference and its 95% confidence interval

Figure 5a evaluates ASES scores at 3 months post-surgery, revealing a wide range of mean differences from − 3.08 to 11.31 [30, 41, 43]. Among the three studies, two demonstrated significant improvements with PRP [41, 43], while Jo et al. notably favoured the control group that did not receive PRP [30]. The overall pooled mean difference was calculated at 4.44, indicating extremely high heterogeneity (I^2^ = 99.92%, *p* < 0.001), which highlights the significant discrepancies in the study results.

Figure 5b focuses on ASES scores at the 6-month mark post-surgery, where the mean differences ranged from − 2.50 to 5.79 [30, 31, 39, 41–43]. Notably, four of the studies indicated significant improvements in favour of PRP treatment [30, 41–43], while Pandey et al. reported results favouring the non-PRP control group [39]. The pooled mean difference observed was 2.69, with extremely high heterogeneity (I^2^ = 99.63%, *p* < 0.001), underscoring the variability in outcomes across the studies.

Figure 5c examines ASES scores at 12 months post-surgery, where mean differences varied from − 1.3 to 3.38 [31, 32, 39]. In this timeframe, two studies found significant improvements with the use of PRP [32, 39], while Rossi et al. showed a preference for the non-PRP control group [31]. The pooled mean difference stood at 1.02, accompanied by extremely high heterogeneity (I^2^ = 99.06%, *p* < 0.001), indicating notable differences in the findings across the research.

Figure 5d analyses ASES scores at 24 months post-surgery, revealing mean differences that ranged from − 0.50 to 5.20 [31, 40, 41]. In this analysis, two studies reported significant improvements associated with PRP treatment [31, 40], contrasting with Flury et al., who favoured the non-PRP group [41]. The pooled mean difference was recorded at 2.17, along with extremely high heterogeneity (I^2^ = 99.54%, *p* < 0.001), reflecting the considerable variability in study outcomes.

### Retear rates

**Fig. 6:**
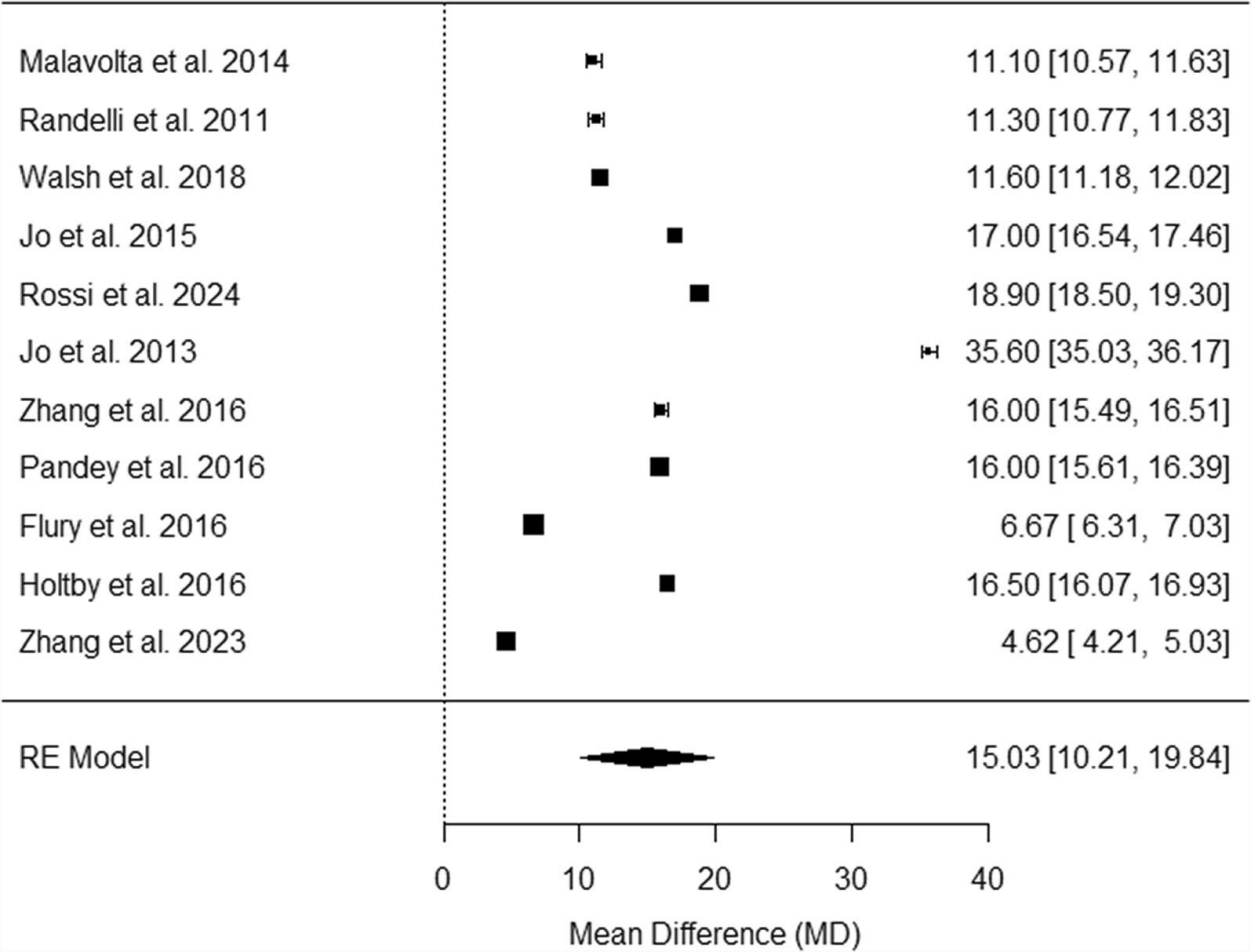
Forest plot of pooled retear rates at 24 months post-surgery. This plot displays the mean differences in retear rates across several studies, each represented by a black square proportional to the weight of the study. Horizontal lines show the 95% confidence intervals for each mean difference. The pooled effect from the random effects model is depicted by a black diamond, indicating the combined mean difference and its 95% confidence interval

Figure 6 provides a comprehensive review of multiple studies assessing mean differences at 24 months post-surgery [24, 26, 28, 30–33, 39, 41–43]. Mean differences are significantly varied, ranging from 4.62 to 35.60, with all studies showing significant improvements in the PRP group [24, 26, 28, 30–33, 39, 41–43]. The random effects model estimates a pooled mean difference of 15.03 [10.21, 19.84], with low heterogeneity (I^2^ = 99.2%, *p* < 0.01).

## Discussion

The findings of this systematic review and meta-analysis shed new light on the role of PRP in enhancing outcomes following rotator cuff surgery. Our analysis demonstrated that PRP significantly reduced postoperative pain in the short term, with a pooled mean difference in VAS scores of 1.22 at 1 month (*p* < 0.01) compared to surgery alone. However, the benefit diminished over time, with non-significant differences observed at 12 months (*p* = 0.47) and 24 months (*p* = 0.06). Similarly, functional outcomes showed initial improvements, particularly in the UCLA Shoulder Rating scores, with a pooled mean difference of 1.89 at 6 months, but these gains were not consistently sustained over longer follow-up periods. Notably, PRP had a significant effect on reducing retear rates, with a pooled risk difference of 15.53 at 24 months. Despite these promising findings, high heterogeneity across studies and variability in PRP preparation highlight the need for standardised protocols and further research to establish its long-term efficacy. Our study thus contributes valuable insights into the short-term advantages of PRP, while underscoring the challenges in demonstrating sustained benefits.

PRP has garnered significant attention regarding its potential in enhancing postoperative recovery [37, 45]. Our study’s analysis of pain outcomes, particularly those captured by the Visual Analogue Scale (VAS), indicated that PRP offers significant short-term benefits. At one month postoperatively, patients receiving PRP demonstrated marked reductions in pain compared to those undergoing surgery alone.

This is consistent with existing literature, which has frequently emphasised PRP's anti-inflammatory properties and its ability to modulate pain pathways in the initial phases of healing [46]. Notably, studies such as those by Randelli et al. and Kesikburun et al. have reported similar short-term analgesic benefits, further corroborating our findings [26, 47]. Randelli et al. conducted a randomised controlled trial on patients undergoing rotator cuff repair, finding that PRP significantly reduced pain in the first six weeks post-surgery [26]. Similarly, Kesikburun et al.’s trial on chronic rotator cuff tendinopathy reported that PRP injections provided marked pain relief within the first three weeks, although the effect tapered off over time [47]. However, the absence of a standardised PRP preparation protocol introduced potential variability in outcomes, while the relatively small sample size and lack of long-term follow-up beyond one year limit the ability to assess the durability of PRP’s effects [47].

However, as demonstrated in our meta-analysis, the magnitude of pain relief diminished over time, with the pooled mean difference in VAS scores dropping to 0.33 at 3 months (*p* = 0.002) and further to 0.29 at 6 months (*p* = 0.021). Beyond this point, the differences became minimal, with non-significant reductions of 0.13 at 12 months (*p* = 0.274) and 0.22 at 24 months (*p* = 0.108), indicating that PRP’s impact on pain relief was largely confined to the early postoperative period. This trend raises critical clinical questions regarding the longevity of PRP’s analgesic effects. Specifically, should PRP be utilised to optimise pain management primarily in the early postoperative phase, where its benefits are most pronounced, rather than relying on it for sustained long-term pain relief? Addressing this requires further investigation into whether modifying PRP preparation, dosing or timing could extend its efficacy. While our findings reinforce PRP’s short-term benefits, they also highlight the need for strategies to improve its durability, potentially reshaping its role in postoperative protocols.

The variability in long-term pain outcomes is likely influenced by differences in the specific protocols for PRP preparation and application across studies. In our meta-analysis, the heterogeneity observed, particularly in pain outcomes at 12 and 24 months, suggests that factors such as the type of PRP used, such as leukocyte-rich versus leukocyte-poor, the concentration of growth factors, the volume of PRP administered and the timing of its application relative to surgery could substantially impact its efficacy. These variations may explain inconsistencies in outcomes such as sustained pain relief, functional recovery and retear rates, highlighting the need for standardised protocols to optimise both short-term and long-term surgical success. This aligns with broader observations in the literature, which have highlighted the need for standardised PRP protocols to mitigate inconsistencies in clinical results [48]. Several studies included in our review applied PRP intraoperatively, while others used it postoperatively with varying degrees of efficacy [29, 34, 35, 37, 44, 49, 50]. This lack of uniformity likely contributes to the mixed findings regarding PRP’s long-term analgesic efficacy and complicates efforts to draw firm conclusions about its sustained clinical benefits [51].

In terms of functional outcomes, our study revealed mixed results. While some functional scores, such as the UCLA Shoulder Rating, showed moderate improvements in patients treated with PRP, these effects were not consistently observed across all time points. Most notably, functional gains were more pronounced at six months, suggesting that PRP may accelerate functional recovery in the medium term. This finding is congruent with the literature, which has frequently reported short- to mid-term functional gains with PRP, particularly in tendon healing [52]. However, the benefits appeared to plateau or diminish beyond 12 months, echoing concerns about the longevity of PRP’s effects. Other investigations have echoed these findings. For example, a study by Randelli et al. found that PRP provided short-term functional benefits, particularly in improving tendon integrity postoperatively, but it showed limited long-term improvement [26]. While they highlighted a statistically significant improvement in functional scores at 3 months between the treatment and control group, no significant difference was observed at 6, 12 or 24 months(25). Other systematic reviews and meta-analysis failed to show any significant improvement in functional outcomes in neither the short-term, mid-term or long-term analysis. For example, a systematic review by Lin et al. found that PRP provided significant pain relief in the long-term, but the functional improvements remained inconsistent [53]. This observation prompts further inquiry into whether PRP’s role in promoting tendon regeneration is time-dependent, with its biological impact potentially waning as tissue remodelling progresses.

Furthermore, a lack of significant long-term functional benefits observed in our study highlights the need for more consistent outcome reporting and a better understanding of which functional measures are most sensitive to detecting changes post-PRP application. As such, a meta-analysis of 18 studies by Hurley et al. reported significant variability in functional outcomes across studies [54]. Similarly, our meta-analysis showed a significant reduction in retear rates in patients treated with PRP, particularly at the 24-month follow-up. This supports evidence suggesting that PRP may enhance tendon healing by promoting collagen deposition and improving the biomechanical properties of repaired tendons. Tang et al. emphasise the role of growth factors in PRP, which regulate recovery from inflammation, stimulate cell proliferation and promote differentiation, all of which are critical for tendon repair processes [55]. Despite these promising outcomes, our analysis revealed high heterogeneity in retear rates across the included studies, suggesting that the benefits of PRP may not be uniformly experienced by all patients. Tear size, tendon quality and patient-specific factors such as age and comorbidities likely influence the effectiveness of PRP in reducing retears. Goldenberg et al. highlight that while PRP holds potential in promoting tendon healing, particularly in smaller tears, evidence from high-quality human studies is still lacking, especially in patients with large or massive tears [9]. Larger, chronic tears, as noted, may present a compromised biological environment that could impede the regenerative capacity of PRP, leading to mixed clinical results. Therefore, the reduction in retear rates observed in our analysis may be more significant in specific patient subgroups, such as younger individuals with smaller tears or those undergoing primary repairs rather than revision surgeries. Tailoring PRP application based on tear size, patient age and the nature of the surgical repair may be essential to maximising its efficacy. This individualised approach aligns with the growing recognition of the need for targeted biologic therapies, rather than a one-size-fits-all strategy, to optimise tendon healing outcomes.

Despite our systematic search and promising findings, this meta-analysis has several limitations that should be acknowledged. Firstly, all the studies included in our review administered PRP intraoperatively. However, there are studies that have explored postoperative PRP administration, which we did not include [6, 14–17, 19]. Consequently, our analysis may not provide a comprehensive understanding of the optimal timing and application of PRP in rotator cuff repair. Additionally, while we included several functional outcome measures such as the UCLA and ASES scores, other relevant scores like the Constant-Murley score or the DASH were not analysed [39]. The omission of these alternative scoring systems may mean that our evaluation of functional outcomes is not fully representative of the breadth of findings in the literature. WORC data were not analysed to do a paucity of studies reporting it. The Risk of Bias assessment highlighted some concerns, particularly in relation to deviations from the intended interventions and missing outcome data. These issues may have introduced bias in the reported outcomes. Finally, in some of the included studies, the patient population was not blinded to the intervention, which could lead to performance bias. Patients who are aware of their treatment may report outcomes differently, potentially skewing the results, particularly in subjective measures such as pain and functional recovery.

## Conclusion

Our findings indicate that while PRP provides short-term benefits in pain relief and functional recovery, its long-term efficacy is uncertain, with diminishing effects beyond 12 months and high heterogeneity in retear rate reductions at 24 months. Although PRP supports early tendon healing, its role in long-term tissue integrity remains unclear, suggesting its benefits may be wane with time. While it aids early postoperative recovery, its long-term impact on pain and function is minimal, requiring careful patient expectations. The observed reduction in retear rates suggests potential long-term benefits, particularly for high-risk patients, though effectiveness varies by tear size, age and comorbidities. Standardising PRP protocols and adopting a patient-specific approach will be key to optimising its clinical application in rotator cuff repair.

## Supplementary Information

Below is the link to the electronic supplementary material.

Supplementary file1 (DOCX 2632 kb)

## Data Availability

No datasets were generated or analysed during the current study.
