# Supplementary material for: Evaluating the longitudinal efficacy of platelet-rich plasma in rotator cuff surgery: a systematic review and meta-analysis
Source: Musculoskelet Surg. 2025 Jul 14;110(1):41–61. doi: 10.1007/s12306-025-00906-9 (PMC12950081; doi:10.1007/s12306-025-00906-9)
Supplement: Supplementary file 1 — Supplementary file1 (DOCX 2632 kb) [file 12306_2025_906_MOESM1_ESM.docx]

Supplementary material

Search string

Embase

| 20 | (biologics or (prp or "Platelet Rich Plasma") or scaffold or ("mesenchymal stem cells" or msp)).mp. and (surgery and "Rotator cuff").m_titl. [mp=title, abstract, heading word, drug trade name, original title, device manufacturer, drug manufacturer, device trade name, keyword heading word, floating subheading word, candidate term word] |
| --- | --- |

Pubmed

| 1448  (67 RCT) | ("biological products"[MeSH Terms] OR ("biological"[All Fields] AND "products"[All Fields]) OR "biological products"[All Fields] OR "biologic"[All Fields] OR "biologicals"[All Fields] OR "biological factors"[MeSH Terms] OR ("biological"[All Fields] AND "factors"[All Fields]) OR "biological factors"[All Fields] OR "biologics"[All Fields] OR "biologically"[All Fields] OR "biology"[MeSH Terms] OR "biology"[All Fields] OR "biological"[All Fields] OR ("pharmacol res perspect"[Journal] OR "prp"[All Fields] OR "Platelet Rich Plasma"[All Fields] OR ("blood platelets"[MeSH Terms] OR ("blood"[All Fields] AND "platelets"[All Fields]) OR "blood platelets"[All Fields] OR "platelet"[All Fields] OR "platelets"[All Fields] OR "platelet s"[All Fields] OR "plateletes"[All Fields]) OR ("plasma"[MeSH Terms] OR "plasma"[All Fields] OR "plasmas"[All Fields] OR "plasma s"[All Fields])) OR ("scaffold"[All Fields] OR "scaffold s"[All Fields] OR "scaffolded"[All Fields] OR "scaffolder"[All Fields] OR "scaffolders"[All Fields] OR "scaffolding"[All Fields] OR "scaffoldings"[All Fields] OR "scaffolds"[All Fields]) OR ("mesenchymal stem cells"[All Fields] OR "MSC"[All Fields])) AND (("surgery"[MeSH Subheading] OR "surgery"[All Fields] OR "surgical procedures, operative"[MeSH Terms] OR ("surgical"[All Fields] AND "procedures"[All Fields] AND "operative"[All Fields]) OR "operative surgical procedures"[All Fields] OR "general surgery"[MeSH Terms] OR ("general"[All Fields] AND "surgery"[All Fields]) OR "general surgery"[All Fields] OR "surgery s"[All Fields] OR "surgerys"[All Fields] OR "surgeries"[All Fields]) AND "Rotator cuff"[All Fields]) |
| --- | --- |

Web of Science

| 876 | ( (Biologics OR (PRP OR "Platelet Rich Plasma" OR Platelet OR Plasma) OR (Scaffold) OR ("mesenchymal stem cells" OR MSC)) AND (Surgery AND "Rotator cuff")) |
| --- | --- |

Scopus

| 1746 | ( biologics OR ( prp OR "Platelet Rich Plasma" ) OR ( scaffold ) OR ( "mesenchymal stem cells" OR msc ) ) AND ( surgery AND ( "Rotator cuff" ) ) AND ( LIMIT-TO ( SUBJAREA , "MEDI" ) OR LIMIT-TO ( SUBJAREA , "HEAL" ) ) AND ( LIMIT-TO ( DOCTYPE , "ar" ) ) AND ( LIMIT-TO ( EXACTKEYWORD , "Controlled Study" ) ) |
| --- | --- |
